# An advection-deposition-survival model to assess the risk of introduction of vector-borne diseases through the wind: Application to bluetongue outbreaks in Spain

**DOI:** 10.1371/journal.pone.0194573

**Published:** 2018-03-22

**Authors:** Eduardo Fernández-Carrión, Benjamin Ivorra, Ángel Manuel Ramos, Beatriz Martínez-López, Cecilia Aguilar-Vega, José Manuel Sánchez-Vizcaíno

**Affiliations:** 1 VISAVET Center and Animal Health Department, Veterinary School, Universidad Complutense de Madrid, Madrid, Spain; 2 MOMAT Research group, IMI-Institute and Applied Mathematics Department, Universidad Complutense de Madrid, Madrid, Spain; 3 CADMS Center for Animal Disease Modeling and Surveillance, School of Veterinary Medicine, UC Davis, Davis, California, United States of America; University of Texas Medical Branch at Galveston, UNITED STATES

## Abstract

This work develops a methodology for estimating risk of wind-borne introduction of flying insects into a country, identifying areas and periods of high risk of vector-borne diseases incursion. This risk can be characterized by the role of suitable temperatures and wind currents in small insects’ survival and movements, respectively. The model predicts the number density of introduced insects over space and time based on three processes: the advection due to wind currents, the deposition on the ground and the survival due to climatic conditions. Spanish livestock has suffered many bluetongue outbreaks since 2004 and numerous experts point to *Culicoides* transported by wind from affected areas in North Africa as a possible cause. This work implements numerical experiments simulating the introduction of Culicoides in 2004. The model identified southern and eastern Spain, particularly between June and November, as being at greatest risk of wind-borne *Culicoides* introduction, which matches field data on bluetongue outbreaks in Spain this year. This validation suggests that this model may be useful for predicting introduction of airborne pathogens of significance to animal productivity.

## Introduction

Introduction of vector-borne diseases can substantially harm public and animal health, causing significant sanitary and financial loss. Long-range wind-borne transportation of infected flying insects has previously been linked to the introduction of viruses affecting humans and/or animals such as West Nile virus, dengue or Rift Valley fever. Therefore, modeling the movement of wind-borne midges or mosquitoes may help identify geographic regions and seasons at higher risk of incursion of several arboviral diseases, improving surveillance efforts.

Bluetongue is a viral disease of ruminants transmitted by biting midges of the genus *Culicoides*. *Culicoides imicola* is considered the main vector of Bluetongue in South Europe and Africa [1]. This virus has traditionally been considered endemic to tropical and subtropical regions, between parallels 35°S and 40°N; however, recently several outbreaks have occurred further North and it is considered to be a re-emerging disease in new latitudes [2]. So far, the disease has been reported in Australia, USA, Africa, Middle East, Asia and Europe, although the geographic distribution of the 27 virus serotypes differs by region [3].

Previous studies suggest that *Culicoides* could be transported by the wind to distances of up to 170 km from their original location [4]. Wind-borne movement of bluetongue virus-infected *Culicoides* is thought to explain the northward spread of bluetongue in Europe, primarily through routes from Turkey to Greece-Bulgaria, from Algeria-Tunisia to Italy and from Morocco-Algeria to Spain [5]. Specifically, wind-borne introduction of these insects to Spain from North Africa has been suggested as the cause of the numerous bluetongue outbreaks in Spain in 2004–05 [6–8] and in other countries and periods [9, 10]. In October 2004, 121 bluetongue outbreaks were reported in Spain; this number increased to 322 in December 2004 and to 328 at the start of 2005 [11–13].

The goal of the present work was to develop a model that could identify geographic areas and time periods at highest risk of introduction of small insects potentially infected with human and/or animal pathogens. The model takes into account three processes: (1) advection, in which insects are considered as particles in the air, and move according to wind currents; (2) deposition, in which wind-borne insects are deposited on the ground; and (3) survival, reflecting insects’ survival or mortality as a function of climatic factors. This model was numerically implemented to simulate wind-borne *Culicoides* introduction from North Africa into Spain and was validated using field data on bluetongue outbreaks reported in Spain in 2004.

## Materials and methods

### Model’s scheme

*Culicoides* are 1–3 mm in size, they weigh less than 1 mg and, under favorable conditions, they can be found as high as 200 m above the ground [14]. Previous studies consider that the long-range transportation of *Culicoides* is likely caused by wind currents at high altitudes [1, 5, 15]. Therefore the model assumes that the movement of small insects in the air shows a similar behaviour to wind-borne small particles [16]. Then, the model takes into consideration the following processes:

(1) Wind advection. Particle movement in the air due to wind currents is computed using an advection partial differential equation (PDE). The same kind of PDE has been used to predict the movements of dust [17, 18], sediments [19, 20], oil [21, 22] or gaseous substances [23, 24].
(2) Vertical deposition. The model assumes that *Culicoides* deposited onto the ground from the air in a manner similar to other airborne particles. Thus, deposition is computed using particles’ sedimentation and flotation theory based on settling principles through the air, adapted here to the specific characteristics of *Culicoides*.
(3) Survival. Dead insects do not pose an introduction risk. Consequently, the model estimates a mortality rate during wind-borne transport on the basis of climatic variables.

### Mathematical model

We consider the spatial domain $\Omega \subset R^{3}$ and time interval [0, *T*]. We denote by Γ_g_ and Γ_s_(*t*) the subsets of the boundary of Ω corresponding, respectively, to the ground and to the source of *Culicoides* entering into Ω at time *t* ∈ [0, *T*]. *C*(*x*, *y*, *z*, *t*) is the spatial and temporal distribution of the number density of insects (i.e. the number of insects per unit volume) at point (*x*, *y*, *z*) ∈ Ω at time *t* ∈ [0, *T*]. Since the number density of insects varies according to wind advection, vertical deposition and survival, the evolution of *C* is governed by the following equations

$$\{\begin{array}{ll} \frac{\partial C}{\partial t}+\text{div}\left(Cw\right)=-\sigma \left(f,C\right) & \text{in}\;\Omega \times \left(0,T\right],\\ C=C_{0} & \text{in}\;\Omega \times \left\{0\right\},\\ C=C_{s} & \text{on}\;\left\{\left(x,y,z,t\right):\left(x,y,z\right)\in \Gamma_{\text{s}}\left(t\right)\right\}, \end{array}$$ (1)

where *C*_0_ is the initial distribution of the number density of *Culicoides* in Ω, *C*_*s*_(*x*, *y*, *z*, *t*) is the number density of insects at point (*x*, *y*, *z*) ∈ Γ_s_(*t*) at time *t* ∈ [0, *T*], **w** = (*w*_*x*_, *w*_*y*_, *w*_*z*_) is the velocity field related to the wind and deposition effects, which satisfies *w*_*z*_ = 0 on Γ_g_ × (0, *T*], and *σ*(**f**, *C*) is the mortality function (i.e. the number of *Culicoides* that die per unit volume and unit time), which depends on *C* and the spatio-temporal distribution of climatic factors **f** associated with survival.

### Model implementation for the case of *Culicoides*

Here, the model formulated above is tailored for studying the case of the introduction of *Culicoides* from North Africa to Iberian Peninsula and Balearic Islands. The model has been fully developed in Matlab.

#### Domain’s discretization

**Fig 1:**
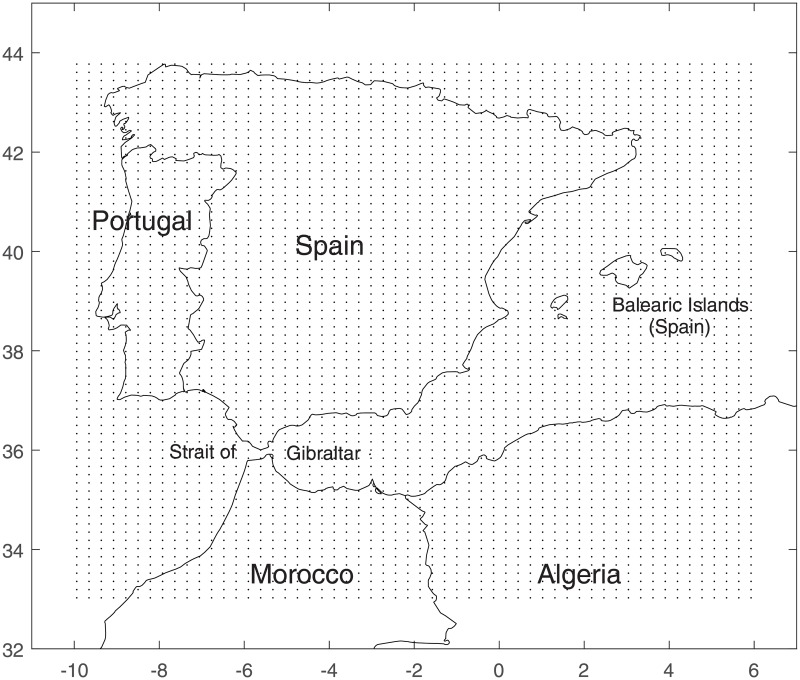
Domain Γ_g_ in numerical experiments, extending from latitude 34°N to 44°N and from longitude 10°W to 4.25°E. Black lines demarcate country boundaries and black dots locate the fixed 2,500 grid points.

We discretized Γ_g_ into a two-dimensional regular meshgrid of *n*_*x*_ × *n*_*y*_ grid points, where $n_{x},n_{y}\in N$. Here we considered *n*_*x*_ = *n*_*y*_ = 50, covering the area extending from latitude 34°N to 44°N and from longitude 10°W to 4.25°E (around 22 and 26 km between vertical and horizontal grid points, respectively), comprising parts of Spain, Portugal, northern Morocco and northern Algeria (Fig 1).

We also discretized the temporal domain [0, *T*] into N∈N steps, using a fixed time step Δ*t* > 0, given by Δ*t* = *T*/*N*. Here, we considered Δ*t* = 1/24 days (1 hour).

#### Climatic data source and interpolation

Wind and temperature data on Γ_g_ were sourced from Forecast.io website (www.forecast.io). This data consisted of hourly measurements of mean temperature (*θ*, in Celsius degrees °C), mean horizontal wind direction (from 0° to 359°) and mean horizontal wind speed (in m/s) taken along 2004 at each grid point on the surface (2,500 source points). Climatic data at intermediate points on the ground within the domain, Γ_g_, were estimated through linear interpolation at every time step.

At high altitudes, *z* > 10 m, temperature values were estimated through the standard free atmospheric lapse rate of 6.5°C decrease per kilometer of increasing elevation estimated for Mediterranean atmosphere [25]:

$$\begin{array}{c} \theta \left(z\right)=\theta \left(z_{0}\right)-\gamma z, \end{array}$$ (2)

where *θ*(*z*_0_) is the temperature on the ground *z*_0_, and *γ* = 0.0065 °C/m.

Horizontal wind speed values at high altitudes, *z* > 10 m, were estimated using the following polynomial variation [26]:

$$\begin{array}{c} w\left(z\right)=w\left(z_{0}\right){\left(\frac{z}{10}\right)}^{\alpha }, \end{array}$$ (3)

where *w*(*z*_0_) is the wind speed on the ground *z*_0_, and *α* is the Hellman exponent for particles above sea and land surfaces (in this work, *α* = 0.10 and *α* = 0.16, respectively).

#### Deposition

Vertical deposition of *Culicoides* from high altitudes was assumed to be governed by processes similar to those affecting the deposition velocity of dust particles. This deposition is due to the combined action of gravitational settling, wind drag capacity and the aerodynamic properties of the particles (here, the *Culicoides*).

In general, flying insects use integrated systems consisting of wings to generate aerodynamic forces, muscles to move the wings, and sensing and control systems to guide and manoeuvre [27]. The Reynolds number, *Re*, and the drag coefficient, *C*_*d*_, are two dimensionless quantities used in fluid mechanics to predict the relative internal movement and drag of particles in different fluid velocities [28]. In particular, as the size of a flier is reduced, the wing-to-body mass ratio tends to decrease paired with the Reynolds number as well [27] (in this work, *Re* = 100, assumed for small flying insects with slow flights [27, 29]). Following Haider et. al. [30], the drag coefficient was computed as a function of the Reynolds number adjusted for small spherical particles:

$$\begin{array}{c} C_{d}=\frac{24}{Re}(1+0.173Re^{0.657})+(\frac{0.413}{1+16,300Re^{-1.09}}). \end{array}$$ (4)

The settling velocity for *Culicoides* was assumed to be governed by the same processes affecting the settling velocity of spherical particles, *V*_*g*_(*z*) [31]:

$$\begin{array}{c} V_{g}{\left(z\right)}^{2}=\frac{8gR}{3C_{d}}(\frac{\rho }{\rho_{\text{air}}\left(\theta \left(z\right)\right)}-1), \end{array}$$ (5)

where *R* is the radius of the *Culicoides*, here assumed to be *R* = 2 ⋅ 10^−3^ m [32]; *M* is the weight of the *Culicoides*, here assumed to be *M* = 0.5 ⋅ 10^−3^ kg [33]; *ρ* is the density of the *Culicoides*, here approximated to an spherical particle *ρ* = *M*/(4/3*πR*^3^); *g* is the gravity acceleration, assumed constant, *g* = 9.81 m/s^2^; and *ρ*_air_(*θ*) is the mass air density at temperature *θ*, see Table 1 [34].

**Table 1:** Effect of temperature on air density.

*θ* (°C)	35	30	25	20	15	10	5	0	-5	-10	-15	-20	-25
*ρ*_air_ (kg/m^3^)	1.15	1.16	1.18	1.20	1.22	1.25	1.27	1.29	1.32	1.34	1.37	1.39	1.42

However, when wind becomes sufficiently strong, particles in the air are susceptible to enter into long-term suspension and are therefore transported long-range [35]. Since one of the main variables for modeling sediment transport is the force exerted on the soil by the wind, a fundamental variable used to predict aeolian particle transport is the shear velocity, ${\vec{u}}_{*}$, whose module is calculated as $u_{*}=\left|{\vec{u}}_{*}\right|=\sqrt{|\vec{\tau }|/\rho_{\text{air}}}$ [36, 37]; where $\vec{\tau }$ is the surface shear stress, i.e. the vertical flux of horizontal momentum measured near the surface; which can be computed as $\vec{\tau }=\rho_{\text{air}}C_{d}w{\left(z_{0}\right)}^{2}$ [38, 39]. Thus,

$$\begin{array}{c} u_{*}^{2}=C_{d}w{\left(z_{0}\right)}^{2}. \end{array}$$ (6)

We may now use the dimensionless parameter $\lambda \left(z\right)=\frac{V_{g}\left(z\right)}{u_{*}}$ to distinguish three types of flight for *Culicoides* [36, 37]: i) a long term suspension, when λ < 0.1; ii) a short term suspension, when 0.1 < λ < 0.7; iii) and vertical settling, when λ > 0.7. Concretely, the settling velocity of *Culicoides*, *w*_*z*_, was computed in terms of *V*_*g*_ (see Eq 5) through the following continuous function:

$$\begin{array}{c} w_{z}\left(z\right)=\{\begin{array}{cc} 0 & \text{if}\;\lambda (z)\leq 0.1,\\ \frac{\lambda (z)-0.1}{0.6}V_{g}\left(z\right) & \text{if}\;0.1<\lambda (z)<0.7,\\ V_{g}\left(z\right) & \text{if}\;\lambda (z)\geq 0.7. \end{array} \end{array}$$ (7)

Since the number density of wind-borne particles is generally greater at lower altitudes than at higher ones [40, 41], we assumed the center of mass of wind-borne living midges to lie at an altitude of 1 km. On the other hand, according to the literature, while long-range transportation of *Culicoides* is caused by wind currents at high altitudes, favorable temperatures and wind speeds for natural *Culicoides* outdoor mobility are often located less than 200 m above the ground [1, 5, 14, 15]. Thus, the model assumes that below this altitude, the *Culicoides* do not move through wind currents (i.e. **w** = (0, 0, 0) if *z* < 200 m).

#### Mortality function

The mortality function predicts the number density of insects based on temperature, since this is one of the most important determinants of *Culicoides* life cycle and lifespan [42, 43]. Although other climatic parameters, such as relative humidity and precipitation, can also affect insect survival, neglecting these factors only moderately affects the accuracy of model predictions [44]. In order to simplify the model, we only considered the impact of temperature in the *Culicoides* mortality function *σ*; i.e. **f** = *θ*, with *θ*(*x*, *y*, *z*, *t*) being the temperature value (here in °C) at point (*x*, *y*, *z*) ∈ Ω and at time *t* ∈ [0, *T*]. Therefore the mortality function is defined by

$$\begin{array}{c} \sigma \left(\theta ,C\right)=\{\begin{array}{cc} \mu (-6)C & \text{if}\;\theta <-6,\\ \mu (\theta )C & \text{if}\;-6\leq \theta \leq 42,\\ \mu (42)C & \text{if}\;\theta >42, \end{array} \end{array}$$ (8)

where *μ* is the *Culicoides* mortality rate (i.e. the inverse of lifespan) as a function of temperature. This function was derived based on studies reporting total *Culicoides* mortality after 2 h at 42°C or −6°C [45] and *Culicoides* lifespan was assumed to be shorter than 24 h after exposure to 40.5°C or −4°C [45]. *Culicoides* lifespan of 27.5, 18.8, 13.4 and 10.2 days were observed at temperature of 10°C, 15°C, 20°C and 30°C, respectively [46]. These values were used to estimate intermediate values for arbitrary temperatures through the Hermite cubic interpolation computed with the Matlab function *pchip* and denoted by 1/*μ*(.). Thus the inverse function *μ*(.) returns the estimated mortality rate of the *Culicoides* depending on the value of the temperature (Table 2).

**Table 2:** Estimated mean lifespan of *Culicoides imicola* at different temperatures, based on field data [45, 46].

Temperature (°C)	-6	-4	15	20	25	30	40.5	42
Lifespan (days)	1/12	1	27.5	18.8	13.4	10.2	1	1/12

#### *Culicoides* sampling data and activity function

Data on the number of *Culicoides imicola* trapped per day along the Spanish territory between 2004 and 2013 and average temperatures associated with each capture were collected from the Ministry of Agriculture, Fisheries, Food and Environment (www.mapama.gob.es) within the Spanish national surveillance programme.

Field data on the mean number of *Culicoides* trapped in Spain at temperatures between 0°C and 35°C were fitted to a polynomial function computed with the *polyfit* command in Matlab, which was defined as the activity function and denoted by *δ*(.). We used this function to estimate the outdoor number density of *Culicoides* in source locations, Γ_s_(*t*).

#### Numerical implementation

The system of Eq (1) was solved numerically using a Lagrangian particle approach. The source of the Lagrangian particles remained constant (i.e. Γ_s_(*t*) = Γ_s_, ∀*t* ∈ [0, *T*]) and was defined as lying in M∈N fixed cells at an altitude of 1 km over northern Morocco and/or northern Algeria. At each time step, *M* new particles started from Γ_s_, such that the model included (*k* + 1) × *M* particles at time step *k* ∈ {0, …, *N* − 1}, and the total amount of particles per simulation was *N* × *M*. All particles were assigned with an index *i* ∈ {1, …, *N* × *M*}. Therefore, at time step *k*, *M* particles with indexes {*k* × *M* + 1, ⋯, (*k* + 1) × *M*} started from Γ_s_.

Particle trajectories were estimated by considering the velocity field **w** through the explicit scheme:

$$\begin{array}{c} X_{n+1}^{i}=X_{n}^{i}+\Delta tw_{n}^{i}, \end{array}$$ (9)

where $X_{n}^{i}$ is the position of the Lagrangian particle with index *i* = {1, …, (*n* + 1) × *M*} at time step *n* = *n*(*i*), ‥, *N*, $w_{n}^{i}$ is the wind velocity vector at position $X_{n}^{i}$ at time step *n* and $n\left(i\right)=\left\lfloor \frac{i-1}{M}\right\rfloor$ (i.e. *n*(*i*) is the greatest integer less than or equal to $\frac{i-1}{M}$). Then, the number density of *Culicoides* transported at step *n* by particle *i* (which was located at $X_{n}^{i}$) was denoted as $C_{n}^{i}$.

Given the difficulty of estimating the number of *Culicoides* placed in a specific location, several epidemiological works assume an initial ratio of mosquitoes or midges instead [47, 48]. In our case, the number density of *Culicoides* transported by particle *i* situated in Γ_s_ at step *n* was assumed to be proportional to the *Culicoides* activity function, *δ*(.), as follows

$$\begin{array}{c} C_{n}^{i}=\delta \left(\theta \left(X_{n}^{i}\right)\right)\text{,}\;\text{if}\;X_{n}^{i}\in \Gamma_{s}. \end{array}$$ (10)

Next, the evolution of this particle number density function was governed by the following scheme:

$$\begin{array}{c} C_{n+1}^{i}=C_{n}^{i}-\frac{\Delta t}{2}(\mu \left(\theta \left(X_{n}^{i}\right)\right)C_{n}^{i}+\mu \left(\theta \left(X_{n+1}^{i}\right)C_{n+1}^{i}\right)), \end{array}$$ (11)

where *i* = {1, …, (*n* + 1) × *M*} and *n* ∈ {*n*(*i*), ‥, *N* − 1}.

According to the features of the *Culicoides*, the number density of *Culicoides* deposited on Γ_g_ was calculated at each time step *n* as the sum of all particles below 200 m altitude; consequently, Eq (11) was not applied to such particles.

#### Simulations

Three numerical experiments were performed to evaluate the risk of *Culicoides* introduction in Spain from North Africa and thereby allow validation of our model against field data of bluetongue outbreaks reported in 2004. The first experiment compared the introduction of *Culicoides* along an entire year from the cells located at 1 km above the ground of Morocco alone, Algeria alone, and both countries (i.e. the choice of three different sources Γ_s_). Each simulation started on the first day of 2004 and finished after *T* = 365 days. The goal of this experiment was to identify areas at high risk of midge introduction during one year from different African countries.

The second experiment examined the risk of wind-borne *Culicoides* introduction from both Morocco and Algeria for each month of a year; thus, 12 simulations were performed, each starting on the first day of every month. The goal of this experiment was to identify months associated with higher risk of *Culicoides* introduction.

A third experiment aimed to study the influence that variation in the values of input parameters affect in the model outcomes, especially the climatic factors and the physical features of the *Culicoides* on the final depositions in the target territory. Thus, a sensitivity analysis was carried out by increasing ±1 to ±5% and ±5 to ±25% the value of the following climatic and physical variables, respectively, in an entire year of simulation from initial *Culicoides* positions located at 1 km above the ground of Morocco and Algeria: temperature, *θ*, wind speed, *w*, Hellman exponent, *α*, temperature lapse rate, *γ*, and insect’s weight, *M*, radius, *R*, Reynolds number, *Re*. The results of the model is also associated to the initial altitude at source locations, Γ_s_, here located at 1 km high. Thus, a sensitivity analysis was carried out by increasing ±5 to ±25% the initial altitude.

The results of the numerical experiments were compared with field data from the bluetongue outbreaks in Spain in 2004. This allowed us to validate the model.

## Results

### Mortality and activity functions

**Fig 2:**
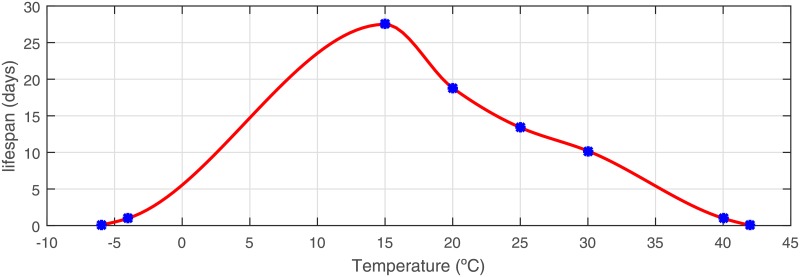
*Culicoides* lifespan used in the present model according to temperature variations based on field data (blue dots) and cubic Hermite approximation (red line), 1/*μ*(.).

Fig 2 shows the lifespan associated to different values of temperature (blue dots) displayed in Table 2 and the cubic Hermite interpolation computed for intermediate values (red line). Thus, the mean lifespan of *Culicoides* is

$$\begin{array}{c} \frac{1}{\mu (\theta )}=\sum_{i=0}^{3}a_{k}\left(\theta \right){\left(\theta -b\left(\theta \right)\right)}^{k}, \end{array}$$ (12)

where the value of *a*_*k*_(*θ*), for each *k* ∈ {0, …, 4}, and *b*(*θ*) are given in Table 3, depending on the value of *θ*.

**Table 3:** Piecewise cubic Hermite interpolating polynomial parameters for the mean lifespan of *Culicoides* according to temperature values from −6°C to 42°C, see Eq (12).

*θ* range (°C)	*a*_0_ (days)	*a*_1_ (days/°C)	*a*_2_ (days/°C^2^)	*a*_3_ (days/°C^3^)	*b* (°C)
-6 ≤ *θ* ≤ -4	1/12	3.69⋅10^−1^	1.48⋅10^−2^	1.49⋅10^−2^	-6
-4 ≤ *θ* ≤ 15	1	6.07⋅10^−1^	1.56⋅10^−1^	-6.04⋅10^−3^	-4
15 ≤ *θ* ≤ 20	27.5	0	-7.77⋅10^−1^	8.59⋅10^−2^	15
20 ≤ *θ* ≤ 25	18.8	-1.33	4.58⋅10^−2^	9.40⋅10^−4^	20
25 ≤ *θ* ≤ 30	13.4	-8.04⋅10^−1^	8.55⋅10^−2^	-1.05⋅10^−2^	25
30 ≤ *θ* ≤ 40.5	10.2	-7.40⋅10^−1^	-6.91⋅10^−2^	5.11⋅10^−3^	30
40.5 ≤ *θ* ≤ 42	1	-5.89⋅10^−1^	7.30⋅10^−2^	-9.06⋅10^−3^	40.5

**Fig 3:**
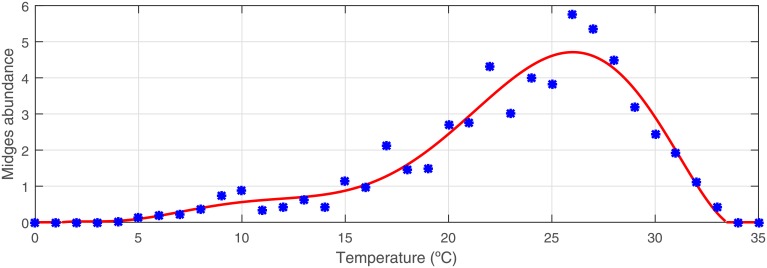
Averaged *Culicoides* trapped per capture (blue dots) and polynomial approximation curve (red line), *δ*(.).

Fig 3 shows the mean number of *Culicoides* captured (blue dots) and the approximation polynomial fitting (red line). Thus, considering a midges abundance higher or equal to zero, the *Culicoides* activity function is defined as

$$\begin{array}{c} \delta \left(\theta \right)=\sum_{i=0}^{8}c_{k}\theta^{k}, \end{array}$$ (13)

where *c*_0_ = −0.226, *c*_1_ = 0.344 °C^−1^, *c*_2_ = −0.178 °C^−2^, *c*_3_ = 4.24 ⋅ 10^−2^ °C^−3^, *c*_4_ = −4.85 ⋅ 10^−3^ °C^−4^, *c*_5_ = 2.89 ⋅ 10^−4^ °C^−5^, *c*_6_ = −9.04 ⋅ 10^−6^ °C^−6^, *c*_7_ = 1.38 ⋅ 10^−7^ °C^−7^ and *c*_8_ = −7.90 ⋅ 10^−10^ °C^−8^, if *θ* ∈ [1.25, 33.45] °C; and *c*_*k*_ = 0 °C^−*k*^, for each *k* ∈ {1, …, 8}, otherwise.

### Annual risk of introduction

**Fig 4:**
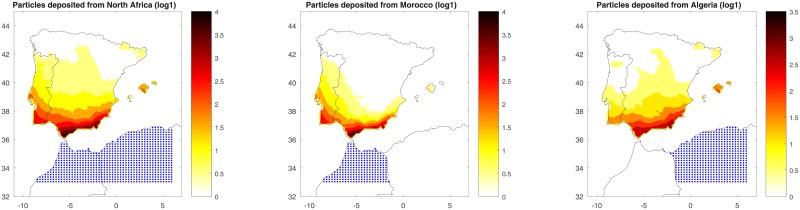
Simulated spatial distribution of the number density of *Culicoides* deposited on the target territory throughout 2004, when the midge source was both Morocco and Algeria (left), Morocco alone (center) or Algeria alone (right). Number densities were plotted in logarithmic scale, *log*(*C* + 1) (log1). Blue dots represent the source of *Culicoides*, Γ_*s*_.

Fig 4 shows the number density of *Culicoides* deposited (in logarithmic scale) across the entire year on target territory via wind-borne transport from the skies over Morocco, Algeria or both. The most exposed areas were the south and east of the Iberian Peninsula and Balearic Islands. Midge deposition patterns varied considerably depending on whether the source was Morocco or Algeria.

When Morocco was the source, midge depositions constituted the 50.99% of the total depositions, and they occurred mainly in the south cost of the Iberian Peninsula, and especially close to the Strait of Gibraltar. However, the *Culicoides* spread might reach the inland west areas of the Iberian Peninsula, reaching even the north of Portugal. On the other hand, a low number density of *Culicoides* reached the Balearic Islands, discarding the introduction of *Culicoides* in the center of the Iberian Peninsula.

When Algeria was the source, midge depositions constituted the 49.01% of the total depositions, and they occurred mainly in the region from southeastern Spain to the eastern coast and Balearic Islands. In some cases, the *Culicoides* spread may reach the center and the northeast of the Iberian Peninsula, but in very low quantity.

### Monthly risk of introduction

Since changes in wind currents and temperature during the year can affect the dynamics of midge transport from North Africa, we simulated the spatial density of deposited *Culicoides* over all 12 months of the year. Table 4 shows the percentage of the number densities of *Culicoides* deposited on the target territory during each month of 2004. Despite having strong wind currents between December and February, the low temperatures do not favor the *Culicoides* activity and it represented a low risk for introduction, reaching 12.65% of the total depositions in a year. Despite favorable temperatures between March to May, the wind currents were not sufficiently strong to transport high amount of *Culicoides*, only reaching 19.40%. The period of high risk of introduction is concentrated in the remaining half of the year, reaching 29.43% between June and August; and 38.52% between September and November, when strong wind streams and suitable temperatures for *Culicoides* activity in African territories are optimal.

**Table 4:** Simulated percentage of number density of *Culicoides* deposited on the ground during each month of 2004.

Jan	Feb	Mar	Apr	May	Jun	Jul	Aug	Sep	Oct	Nov	Dec
5.21	1.44	5.43	9.70	4.27	7.50	15.11	6.81	9.33	15.33	13.85	6.00

**Fig 5:**
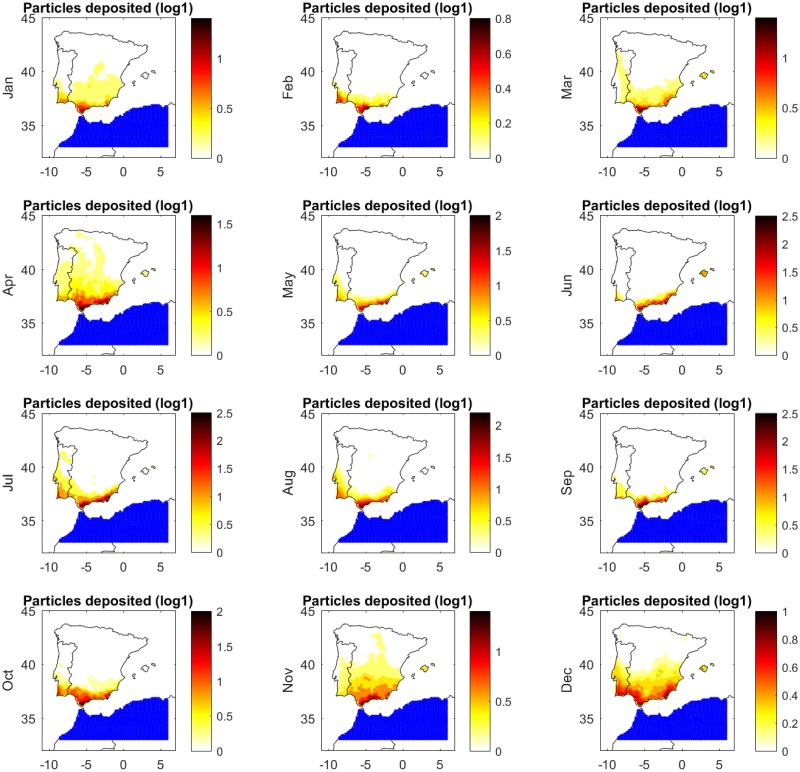
Simulated spatial distribution of the number density of *Culicoides* deposited on Spanish territory for each month of 2004, when the midge source was both Morocco and Algeria. Number densities were plotted in logarithmic scale, *log*(*C* + 1) (log1). Blue dots represent the source of *Culicoides*, Γ_*s*_.

Next we examined the spatial distribution of depositions over the 12 months of the year. Fig 5 shows the monthly distribution of *Culicoides* number density deposited on target territory from the source in both Morocco and Algeria. The largest densities of deposition occurred in the southern and eastern parts of the Iberian Peninsula and Balearic Islands, consistent with the annual-level simulation. Risk of wind-borne incursions were high throughout the year in the southern part of the Iberian Peninsula, specifically close to the Strait of Gibraltar. Central areas of the Iberian Peninsula and the north of Portugal were scarcely reached in specific periods with strong wind streams. However, the Balearic Islands were reached frequently with a high amount of depositions, as mentioned above, mainly from Algeria but also could be reached from Morocco.

### Sensitivity analysis

Table 5 shows the changes in the output number density deposited over target territory, under the assumption of slight variations in climatic variables and in physical features of the *Culicoides*. Among all climatic variables computed, the wind speed and the Hellmann exponent, both associated directly with wind speed at higher altitudes, represented the most relevant variables in terms of particle transportation. Indeed, an increase/decrease of 5% in the wind speed (e.g. from 20 m/s to 21 m/s) may increase/decrease the final number density around ±10%. In contrast, variables affecting temperature slightly varied the final number density. Indeed, a temperature increase/decrease of 5% (e.g. from 20°C to 21°C) may increase/decrease the final number density around ±3%.

**Table 5:** Percentage of output variation of final number density of *Culicoides* deposited over target territory, *C*, after a whole year simulation for input variation of {±5%, ±4%, ±3%, ±2%, ±1%} for climatic variables and {±25%, ±20%, ±15%, ±10%, ±5%} for insect’s variables.

**Percentage of variation**	-5%	-4%	-3%	-2%	-1%	+1%	+2%	+3%	+4%	+5%
Temperature, *θ*	-3.65	-2.86	-1.89	-1.14	-0.35	0.98	1.52	2.01	2.40	2.84
Wind speed *w*	-9.07	-7.18	-5.23	-3.43	-1.60	2.14	4.12	6.04	8.01	9.98
Hellman exponent, *α*	-5.89	-4.75	-3.65	-2.40	-0.84	1.76	3.16	4.48	5.95	7.36
Temperature lapse rate, *γ*	2.15	1.69	1.25	0.75	0.28	-0.22	-0.85	-1.38	-1.63	-2.02
**Percentage of variation**	-25%	-20%	-15%	-10%	-5%	+5%	+10%	+15%	+20%	+25%
Insect’s weight, *M*	10.09	7.99	5.88	4.02	2.13	-1.11	-2.64	-4.22	-5.73	-7.27
Insect’s radius, *R*	-18.90	-14.84	-10.77	-6.80	-2.82	3.65	6.82	10.01	12.69	15.49
Reynolds number, *Re*	8.54	6.87	5.17	3.50	1.80	-3.27	-4.55	-5.80	-7.27	-8.79
**Percentage of variation**	-25%	-20%	-15%	-10%	-5%	+5%	+10%	+15%	+20%	+25%
Initial source altitude, 1000 m	-16.22	-11.68	-8.74	-5.75	-2.43	2.74	4.93	7.15	9.06	11.01

A reduction in the weight of the *Culicoides*, or an increment in the radius decreases the density of the wind-borne particle, *ρ*, which reduces the settling velocity accordingly to Eq 5. On the contrary, a reduction in Reynolds number increases the drag coefficient, *C*_*d*_, which decreases the settling velocity, see Eqs 4 and 5. These cases implied a reduction in settling velocity, which extends long-suspension in the air reaching longer distances.

### Model validation

**Fig 6:**
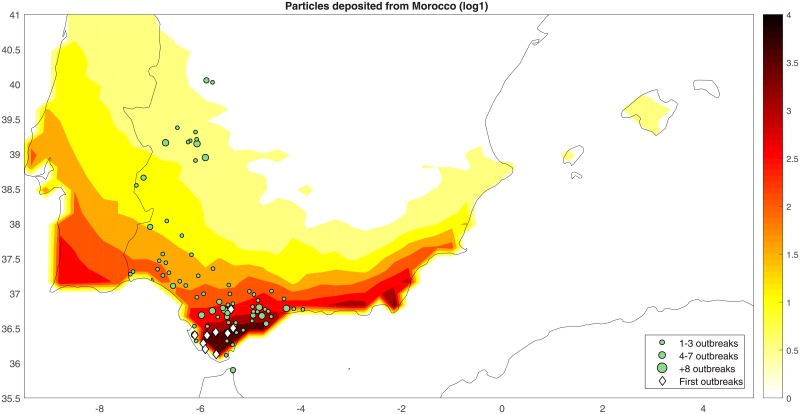
Bluetongue outbreaks reported in Spain in between October 2004 and January 2005 (green dots). First outbreaks reported between October 12th and 19th (white diamonds). Simulated spatial distribution of the number density of *Culicoides* deposited on the Iberian Peninsula throughout 2004 when the midge source was the sky over Morocco (see Fig 5), plotted in logarithmic scale, *log*(*C* + 1) (log1).

Since October 2004 to January 2005, a total of 328 bluetongue outbreaks were reported in the Iberian Peninsula, with no previous outbreaks reported since 1960 [6, 11–13]. Among them, 82% were located in the south of Spain, and only 18% were located in the south-west. Since September 2004 to December 2004, a total of 230 outbreaks were reported in Morocco, with no previous outbreaks reported it that year. Concretely, 28 new outbreaks were reported in September in north-northeastern Morocco. Other works consider that a period of 4 weeks is the time necessary to detect an infected animal once the infected vector lands in the surroundings [15, 49]. According to this, the virus could have been introduced in September from infected areas of Morocco, to the Spanish territory, concretely to areas close to the Strait of Gibraltar (see Fig 6).

## Discussion

In this work, we developed a methodology for estimating risk of long-range wind-borne transport and deposition of small insects, while taking into account the likelihood of insect survival given certain climatic conditions. The methodology relies on a mathematical advection model that is solved numerically using Lagrangian particle tracking, based on the wind velocity field, and particles’ sedimentation and flotation theory for deposition, that takes into account temperature-based insect lifespan and activity. In particular, we focused on assessing the risk of introduction of *Culicoides* from North Africa to Spain, in order to explain the bluetongue outbreaks that occurred in this territory during 2004.

Two numerical experiments were carried out to simulate the incursion of *Culicoides* through wind currents in 2004 in Spain, one yearly and one monthly. The results showed that the highest number density of *Culicoides* deposited on regions agreed with the real primary bluetongue reported outbreaks in the period of study. Concretely, since September 2004, several bluetongue outbreaks were reported in Morocco. In this period, the model showed that main incursions of *Culicoides* from Moroccan territory were deposited close to the Strait of Gibraltar, showing a high rate of matching between model results and bluetongue notifications.

For the remaining reported outbreaks and for future reported ones in 2005 [13], Calvete et al. [50] identified suitable areas for *Culicoides* based on climatic factors and livestock distribution in Spain. Those regions were distributed mainly in the south and south-west of Spain. The model developed in this work aimed to assess midges’ incursions from North Africa due to wind currents, not the spread within the Iberian Peninsula. In future works, this model should link continuous updated bluetongue status in North African areas, with the host population distribution and with spatio-temporal suitable conditions for vector survival in target, as proposed by Calvete et al. [50], in order to estimate not only the risk of introduction, but also the risk of spread.

Our results suggest that the *Culicoides* source can substantially affect midge deposition in Spain: when midges came from over northern Morocco, they usually deposited close to the Strait of Gibraltar; when they came from over northern Algeria, they usually deposited along southeastern Spain and the Balearic Islands. Our results further showed risk variation during the year: risk of introduction was relatively high between June and November, and moderate during other months. These findings illustrate the power of our approach for helping to focus surveillance efforts in space and time.

This work was motivated because, first, different authors foretold the Strait of Gibraltar as one of the main bluetongue introduction pathways in Europe [2, 5] and, second, the presence of *Culicoides* and bluetongue outbreaks were reported along the region and period of study, which allowed the validation of our predictions [11, 12]. The use of the methodology presented here may serve to assess regions and periods at high risk of incursion of several vectors such as midges or mosquitoes. Indeed, *Culicoides* may also be a vector for African horse sickness, and many species of mosquitoes are vectors for diseases such as West Nile virus, dengue, Rift Valley fever, Zika, etc. Currently, the system developed in this work embeds a wind and climatic databases automatically, which allows surveillance of real-time (hourly) wind patterns and *Culicoides* deposition under friendly configurable features, which are easily adaptable to other insects. However, an important quantitative validation of this model should be carried out with more bluetongue outbreaks in territories with no previous disease notifications in a long period; e.g. the bluetongue outbreaks in other European territories or possible infections of African territories from Spanish source locations. The application of this system is to launch risk alerts when suspicious initial infected areas that may spread the vector to long-range distances, especially where livestock population may be exposed. This development could contribute to an effective early warning system for preventing and controlling vector-borne disease incursions.

## Supporting information

S1 File Video simulation. Video file of particle movement in the air and ground concentration on Spanish territory during September 2004.
(MP4)
